# Co-infections exacerbate inflammatory responses in COVID-19 patients, promoting coagulopathy and myocardial injury, leading to increased disease severity

**DOI:** 10.3389/fimmu.2025.1522313

**Published:** 2025-02-19

**Authors:** Xiaoxia Chang, Yanjun Lai, Yingying Zhao, Jing Zhao, Yunchao Zhang, Xiaotao Qian, Guochao Zhang

**Affiliations:** ^1^ Department of Clinical Laboratory, Ninth Hospital of Xi’an, Xi’an, Shannxi, China; ^2^ Department of Pathology, Fenyang College of Shanxi Medical University, Fenyang, Shanxi, China; ^3^ Department of Nephrotic Hemodialysis Center, Shannxi Provincial People’s Hospital, Xi’an, Shannxi, China

**Keywords:** SARS-CoV-2, COVID-19, co-infections, inflammatory response, coagulation dysfunction, myocardial injury

## Abstract

**Objectives:**

Severe COVID-19 infection is characterized by excessive inflammatory responses, hypercoagulation, and microvascular dysfunction. However, limited research has investigated the effects of co-infections on these characteristics in COVID-19 patients. This study aims to explore how co-infections influence inflammation, hypercoagulability, and microvascular dysfunction in hospitalized COVID-19 patients, and to assess their impact on disease progression.

**Methods:**

This was a retrospective cohort study involving 630 COVID-19 inpatients who tested positive for SARS-CoV-2 RNA at Xi’an Ninth Hospital. The patients were categorized into two groups: a severe group (n = 176) and a mild group (n = 454). Additionally, they were further subdivided into a co-infected (n = 106) group and a non-co-infected group (n=524) based on the presence or absence of co-infections. Clinical characteristics and laboratory findings were analyzed and compared between the groups.

**Results:**

In the co-infected group, 60 patients (56.6%) were classified as severe cases, and 15 (14.2%) died. By comparison, in the non-co-infected group, 97 patients (18.5%) were severe cases, with 4 (0.8%) deaths. The severity and mortality rates were significantly higher in co-infected patients compared to those non-co-infections. The severe and co-infected groups exhibited significantly higher levels of inflammatory cells, inflammatory factors, coagulation biomarkers, and myocardial injury markers compared to the mild and non-co-infected groups. Conversely, lymphocyte counts, RBC counts, HGB, HCT, TP, and ALB levels were significantly lower in the severe and co-infected groups than in the mild and non-co-infected groups. Furthermore, a notable positive correlation was observed among inflammatory factors, coagulation function, and myocardial injury biomarkers in COVID-19 patients.

**Conclusion:**

Co-infections in COVID-19 patients can trigger severe inflammatory responses. This excessive inflammation may lead to coagulation disorders and myocardial injury, all of which are key contributors to disease progression and deterioration. Therefore, implementing infection prevention measures to minimize the spread of co-infections among hospitalized COVID-19 patients is crucial.

## 1 Introduction

Novel coronavirus pneumonia (COVID-19) is an acute respiratory illness with high transmissibility, caused by severe acute respiratory syndrome coronavirus 2 (SARS-CoV-2) (1). First identified in December 2019 in Wuhan, China, COVID-19 rapidly spread globally. As of July 14, 2024, the World Health Organization reported over 775 million cases worldwide, with more than 7.05 million deaths (2). Statistics indicate that secondary and opportunistic infections are especially common in severe COVID-19 patients. These include co-infections with pathogens such as influenza viruses and bacteria, particularly among those requiring respiratory support or intensive care (3). Early studies suggest a clear link between bacterial or viral co-infections and more severe clinical outcomes during pandemics (4, 5). Bacterial infections, particularly those caused by *Streptococcus pneumoniae*, *Staphylococcus aureus*, and fungi, are well-known complications of influenza-induced pneumonia. Consequently, this raises questions about whether similar co-infections worsen COVID-19 progression and how they affect disease outcomes (6).

Severe COVID-19 infection is characterized by excessive inflammatory responses, a hypercoagulable state, and microvascular dysfunction (7, 8). In COVID-19, dysregulated immune responses can cause excessive cytokine production, leading to widespread inflammation, commonly known as a ‘cytokine storm.’ This condition can drive hypercoagulability and multi-organ dysfunction. In severe cases, it may progress to multi-organ failure, especially affecting the cardiovascular system (9, 10). Acute inflammation may trigger ventricular arrhythmias, cardiogenic shock, and even death, making cardiovascular complications a recognized feature of severe COVID-19 (11). Remarkably, even patients without pre-existing cardiovascular disease (CVD) may develop abnormalities such as myocardial injury, arrhythmias, and thrombotic events (12). Therefore, cardiovascular complications not only heighten the risk of adverse outcomes but also serve as direct manifestations of COVID-19. Research also shows that COVID-19 can cause coagulation disorders, heightening the risk of thrombosis and contributing to multi-organ failure, including heart failure. The coagulation process entails the activation of a protein cascade involving thrombin and fibrinogen. Conversely, the fibrinolytic system breaks down clots, generating fibrin degradation products. Elevated D-Dimer levels are frequently observed in COVID-19 patients and are strongly correlated with disease severity and mortality risk (13).

**Figure 1:**
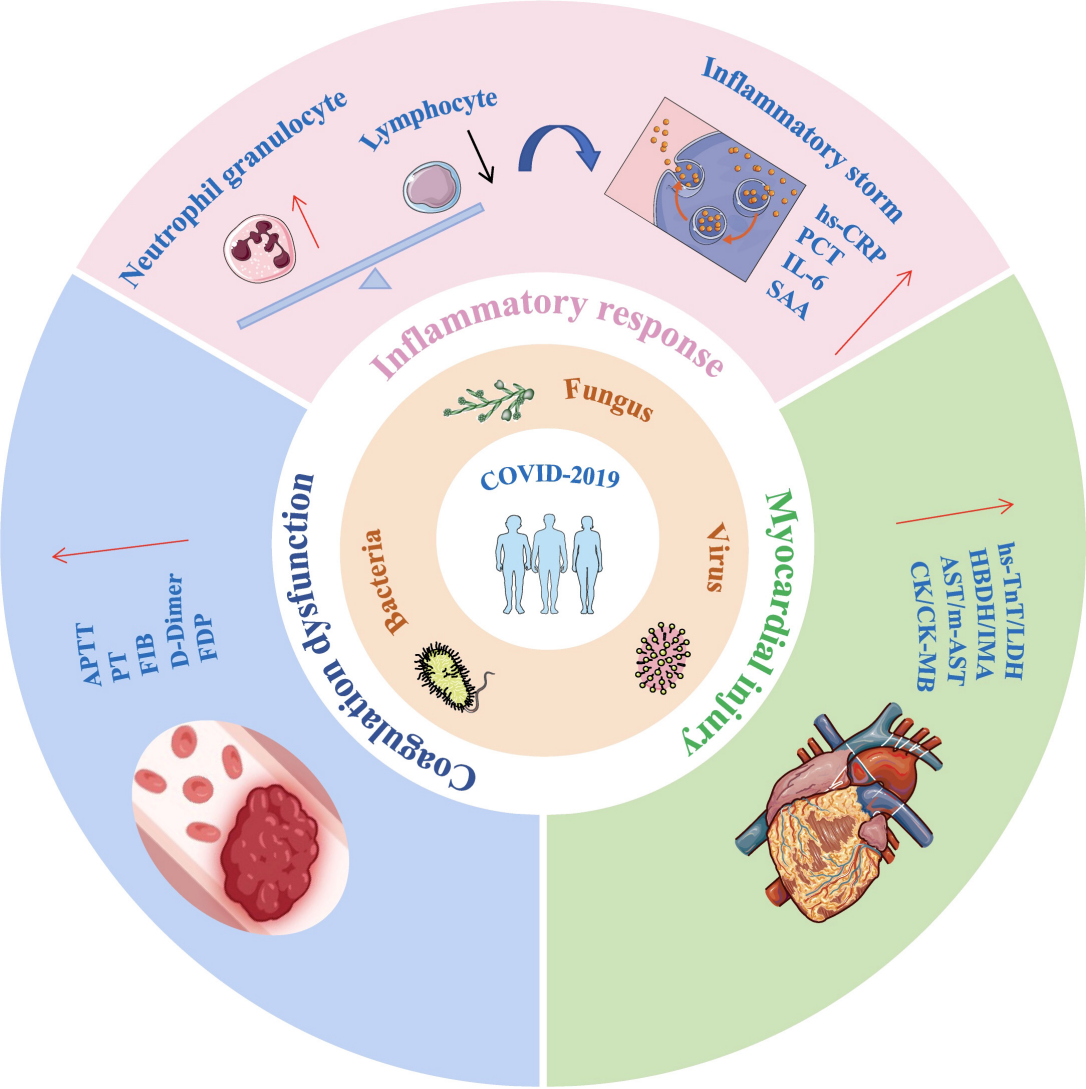
Impact of co-infections on inflammatory response, coagulation function, and myocardial injury in COVID-19 patients. Co-infections in COVID-19 patients can lead to lymphopenia and neutrophilia, triggering a cytokine storm. This is accompanied by significant elevations in coagulation function markers and cardiac injury biomarkers, all of which are critical factors in disease progression and worsening outcomes. IL-6, interleukin-6; hs-CRP, Hypersensitivity C-reactive protein; SAA, serum amyloid A; PCT, Procalcitonin; APTT, activated prothrombin time; PT, prothrombin time; FIB, fibrinogen; FDP, Fibrinogen degradation products; hs-TnT, Hypersensitive cardiac troponin; LDH, lactic dehydrogenase; HBDH, α Hydroxybutyrate dehydrogenase; IMA, Ischemic modified albumin; AST, aspartate aminotransferase; m-AST, mitochondrialaspartate aminotransferase; CK, creatine kinase; CK-MB, Creatine kinase isoenzyme MB.

Extensive research has examined the inflammatory response in COVID-19 and its isolated effects on coagulation dysfunction and myocardial injury. However, the combined impact of co-infection, inflammation, coagulation, and myocardial dysfunction on disease progression remains underexplored. This retrospective study compared the clinical characteristics and laboratory findings of mild and severe COVID-19 patients, as well as those with and without co-infections. The study analyzed changes in inflammatory markers, coagulation parameters, and myocardial injury biomarkers to facilitate early identification and timely intervention for critically ill patients (**Figure 1**).

## 2 Materials and methods

### 2.1 Study design, patient, and clinical data collection

This retrospective cohort study analyzed 630 patients who tested positive for SARS-CoV-2 RNA and were hospitalized at Xi’an Ninth Hospital (Shaanxi, China) from December 10, 2022 to December 31, 2023. COVID-19 diagnoses and classifications followed the “Diagnosis and Treatment Protocol for Novel Coronavirus Infection (Trial Version 10)” issued by China’s National Health Commission (14). Patients exhibited typical COVID-19 symptoms, such as fever and cough, and were confirmed positive via RT-PCR, which differentiated them from other respiratory infections or non-infectious conditions. Excluding hospitalized patients with chronic inflammatory diseases, autoimmune diseases, immunodeficiency, organ transplants, malignant tumors, hematological disorders, or coagulation abnormalities as well as myocardial injury caused by diabetes or heart disease prior to admission, and those with incomplete data. Patients were categorized as mild, moderate, severe, or critical based on clinical symptoms, imaging, and respiratory status. For analysis, mild and moderate cases were grouped as the ‘mild group,’ while severe and critical cases formed the ‘severe group.’ Additionally, patients were categorized into ‘co-infected’ and ‘non-co-infected’ groups based on the presence or absence of co-infections.

Data were collected on demographics (gender, age, illness duration, and medical history), laboratory results (including blood cell counts, inflammatory markers, coagulation parameters, and myocardial injury indicators), and co-infections. Data extraction and verification were performed by two independent researchers. The study was approved by the Institutional Review Board of Xi’an Ninth Hospital (NO.202311) and conducted in accordance with the Declaration of Helsinki. Given its retrospective design, the requirement for informed consent was waived.

### 2.2 Detection of peripheral blood cell counts, inflammatory factors, protein concentrations, coagulation function, and biomarkers of myocardial injury

Blood samples were collected within ≤48 hours after the clinical diagnosis of COVID-19. Peripheral blood was collected in EDTA anticoagulant tubes for hematological analysis using the Mindray CAL8000 blood cell analyzer. Serum was collected in separation gel tubes, with hypersensitivity c-reactive protein (hs-CRP) and serum amyloid A (SAA) measured by the Siemens BN II analyzer. Interleukin-6 (IL-6), procalcitonin (PCT), and hypersensitive cardiac troponin (hs-TnT) levels were analyzed using the Roche Cobas e411 electrochemiluminescence immunoassay system. Serum proteins and myocardial enzymes, including total protein (TP), albumin (ALB), aspartate aminotransferase (AST), mitochondrialaspartate aminotransferase (m-AST), creatine kinase, creatine kinase isoenzyme MB (CK-MB), lactic dehydrogenase (LDH), α Hydroxybutyrate dehydrogenase (HBDH), and ischemic modified albumin (IMA), quantified with the TBA-FX8 biochemical analyzer. Additional samples in sodium citrate anticoagulant tubes were used to assess prothrombin time (PT), activated prothrombin time (APTT), fibrinogen (FIB), D-Dimer (DD), and fibrinogen degradation products (FDP) levels with the Stago-Max.

### 2.3 Microbial cultivation, pathogen identification, and antimicrobial susceptibility testing

Microbiological culture specimens included blood, sputum, and urine samples. Blood specimens were inoculated into both aerobic and anaerobic culture bottles and incubated using the BacT/ALERT 3D automated system. Upon bacterial growth, broth cultures were inoculated onto specific media to isolate pathogens. Deep sputum and midstream urine samples were cultured on blood agar, MacConkey agar, and chocolate agar for pathogen isolation. Pathogen identification was conducted with the VITEK2-compact system and Bruker MALDI-TOF mass spectrometer. Strain isolation, suspension preparation, and antimicrobial susceptibility testing were performed using various methods, including instrumental techniques, the Kirby-Bauer method, and the E-test, with results interpreted according to CLSI M100 standards (2021) (15).

### 2.4 Statistical analysis

Graphpad Prism version 9.5 (Graphpad, LaJolla, CA, USA) and Origin version 2021 (Origin, OriginLab Corporation, USA) were utilized for graphical representation and statistical analysis. For the comparison of continuous data between groups, independent samples t-tests were employed, while categorical data were expressed as frequencies and percentages, with group comparisons performed by chi-square tests. Spearman’s rank correlation coefficient was used to analyze the correlation between the two sets of variables, with a two-tailed α level of less than 0.05 considered statistically significant.

## 3 Results

### 3.1 Basic information of patients with COVID-19

**Figure 2:**
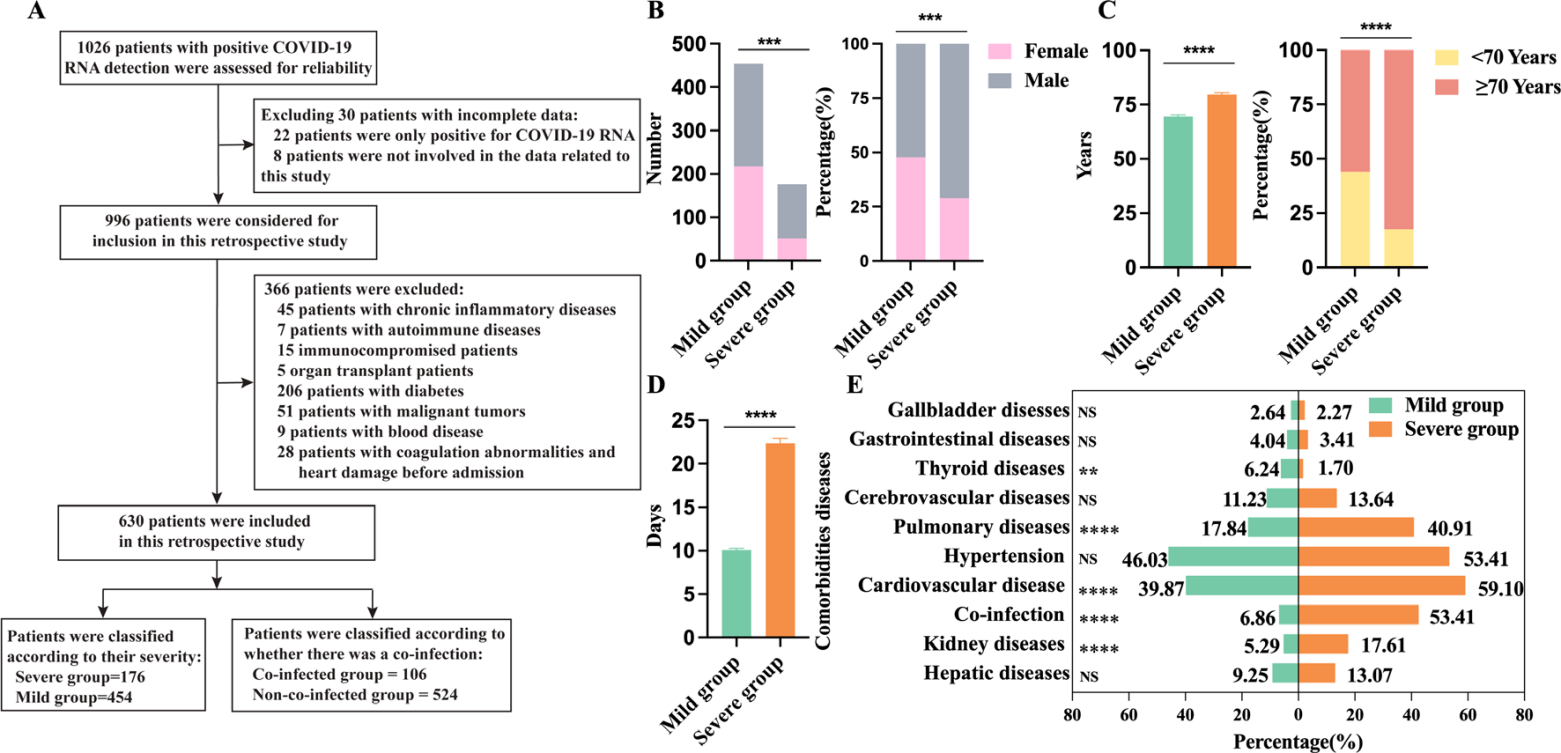
Demographics and clinical characteristics of 630 patients with COVID-19. **(A)** Flowchart of patient inclusion and exclusion; **(B)** Comparison of male and female counts and percentages between the severe group (n=176) and the mild group (n=454); **(C)** Age and percentage distribution comparison between the severe group (n=176) and the mild group (n=454); **(D)** Days from onset to discharge comparison between the severe group (n=176) and the mild group (n=454); **(E)** Comparison of underlying conditions between the severe group (n=176) and the mild group (n=454). n: Total number of patients with available data. P values comparing the severe group and the non-severe group are from χ2 test and unpaired t test. P<0.05 was considered significant. *P<0.05, **P<0.01, ***P<0.001, ****P<0.0001; NS, not significant.

Based on the inclusion and exclusion criteria, a total of 630 hospitalized COVID-19 patients were included in this study (**Figure 2A**), divided into 454 mild cases (52.20% male, 47.80% female) and 176 severe cases (71.02% male, 28.98% female), with 20 total deaths (3.2%), the severe group had a higher proportion of males (**Figure 2B**) and was generally older, averaging 79.70 ± 10.92 years, with a notably higher proportion of patients aged ≥70 (**Figure 2C**). Time from symptom onset to discharge was longer in severe cases (22.35 ± 7.81days) than in mild cases (10.1 ± 3.63 days) (**Figure 2D**). Hypertension (48.10%) was the most common comorbidity, and the severe group had higher rates of pulmonary diseases, cardiovascular disease, and kidney disease (**Figure 2E**).

In addition, we observed that 106 out of 630 patients with COVID-19 (16.8%) experienced co-infections, while 524 cases (83.2%) were only infected with SARS-CoV-2. Among those infected solely with SARS-CoV-2, 423 cases (80.73%) were mild and 101 cases (19.27%) were severe. Among co-infected patients, 75 cases (70.75%) were severe and 31 cases (29.24%) were mild. Co-infections significantly increased the risk of severe symptoms. In the mild co-infected group, 30 cases (96.77%) had single co-infections, while 1 case (3.23%) had mixed co-infections. In severe co-infected patients, 55 cases (73.33%) had single-pathogen infections, and 20 cases (26.67%) had mixed infections. These findings manifest that the risk of severe symptoms increases with multiple pathogen infections. Severe co-infections included 11 Gram-positive, 63 Gram-negative, 11 fungal, and 2 viral cases, while mild co-infections included 4 Gram-positive and 14 Gram-negative bacterial infections, and 3 viral infection. Detailed co-infection characteristics are provided in **Table 1**.

**Table 1:** Findings of the 106 patient s with COVID-19 detected to have other pathogens co-infections.

Infection category	Mild group(n=31)	Severe group(n=75)	*P* value
Mixed co-infection (%)	1 (3.23)	20 (26.67)	0.006
Single co-infection (%)	30 (96.77)	55 (73.33)	0.006
Candida albicans (%)(G^+^)	0	9 (12)	0.098
Candida glabrata (%)(G^+^)	0	1 (1.33)	0.518
Candida krusei (%)(G^+^)	0	1 (1.33)	0.518
Staphylococcus aureus (%) (G^+^)	2 (6.45)	6 (8)	0.784
Staphylococcus hominis (%) (G^+^)	0	1 (1.33)	0.518
Enterococcus faecium (%) (G^+^)	0	1 (1.33)	0.518
Streptococcus pneumoniae (%) (G^+^)	1 (3.23)	2 (2.67)	0.875
Acinetobacter baumannii(%)(G^-^)	1 (3.23)	17 (22.67)	0.015
Klebsiella pneumoniae(%)(G^-^)	6 (19.35)	18 (24)	0.603
Klebsiella oxytoca(%)(G^-^)	0	1 (1.33)	0.518
Escherichia coli(%)(G^-^)	0	9 (12)	0.044
Pseudomonas aeruginosa(%)(G^-^)	4 (12.90)	13 (17.33)	0.572
Burkholderia cepacia(%)(G^-^)	0	1 (1.33)	0.518
Achromobacter xylooxidans (%)(G^-^)	0	1 (1.33)	0.518
Enterobacter cloacae (%)(G^-^)	1 (3.23)	3 (4)	0.879
Mycobacterium tuberculosis	2 (6.45)	0	0.026
Stenotrophomonas maltophilia (%)(G^-^)	1 (3.23)	0	0.118
Corynebacterium striatum(%) (G^+^)	1 (3.23)	1 (1.33)	0.515
Legionella pneumophila (%)(G^-^)	1 (3.23)	0	0.118
Mycoplasma pneumoniae	6 (19.35)	0	<0.0001
Influenza A virus	1 (3.23)	1 (1.33)	0.515
Human metapneumovirus	1 (3.23)	1 (1.33)	0.515
Influenza B virus	1 (3.23)	0	0.118

Data are n (%), where n is the total number of patients with available data. *P* values are from a χ^2^ analysis, *P*<0.05 was considered significant.

G^+^, Gram positive; G^-^, Gram negative;

### 3.2 Changes in inflammatory cells and factors in COVID-19 patients

**Figure 3:**
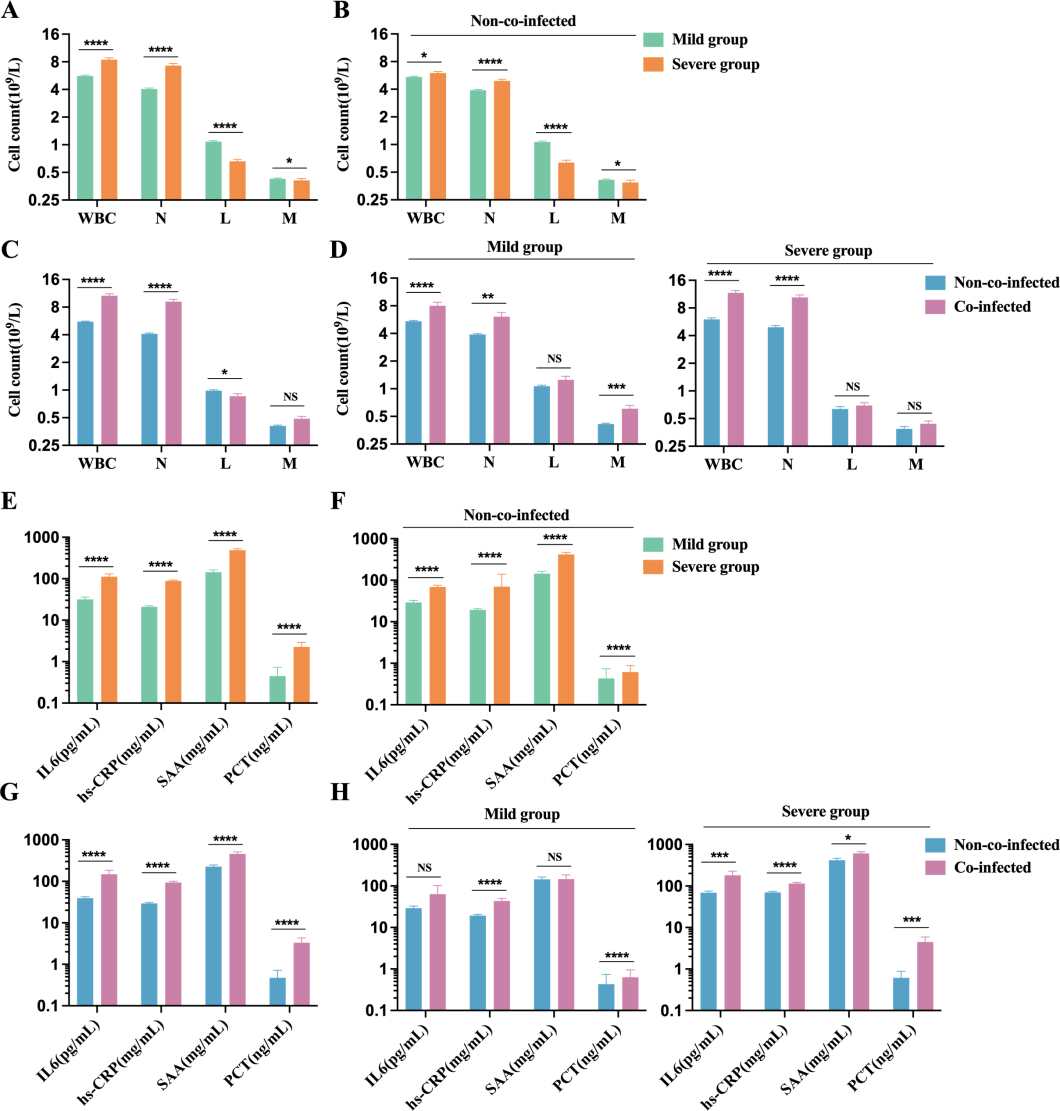
Comparison of inflammatory cell and factors in peripheral blood of patients with COVID-19. Count of inflammatory cells: **(A)** Severe group (n=176) VS. Mild group (n=454); **(B)** Severe group (n=101) VS. Mild group (n=423) in non-co-infected patients; **(C)** Co-infected group (n=106) VS. Non-co-infected group (n=524); **(D)** Non-co-infected VS. Co-infected patients in the mild (co-infected: n=31, non-co-infected: n=423) and the severe groups (co-infected: n=75, non-co-infected=101), respectively. Inflammatory factors: **(E)** Severe group (n=117 for IL-6, n=144 for hs-CRP, n=79 for SAA, and n=151 for PCT) VS. Mild group (n=233 for IL-6, n=439 for hs-CRP, n=132 for SAA, and n=330 for PCT); **(F)** Severe group (n=73 for IL-6, n=101 for hs-CRP, n=50 for SAA, and n=87 for PCT) VS. Mild group (n=216 for IL-6, n=409 for hs-CRP, n=118 for SAA, and n=302 for PCT) in non-co-infected patients; **(G)** Co-infected group (n=61 for IL-6, n=103 for hs-CRP, n=43 for SAA, and n=92 for PCT) VS. Non-co-infected group (n=289 for IL-6, n=510 for hs-CRP, n=168 for SAA, and n=389 for PCT); **(H)** Non-co-infected VS. Co-infected patients in the mild(co-infected: n=14 for IL-6, n=30 for hs-CRP, n=14 for SAA, and n=28 for PCT; non-co-infected: n=216 for IL-6, n=409 for hs-CRP, n=118 for SAA, and n=302 for PCT) and the severe group (non-co-infected: n=44 for IL-6, n=73 for hs-CRP, n=29 for SAA, and n=64 for PCT; non-co-infected: n=73 for IL-6, n=101 for hs-CRP, n=50 for SAA, and n=87 for PCT), respectively. n: Total number of patients with available data. All data presented as the mean ± SEM. Differences were tested using unpaired t test. P<0.05 was considered significant. *P<0.05, **P<0.01, ***P<0.001, ****P<0.0001. NS, not significant. WBC, White blood cell count; N, Neutrophil count; L, Lymphocyte count; M, Monocyte Count; IL-6, interleukin-6; hs-CRP, Hypersensitivity C-reactive protein; SAA, serum amyloid A; PCT, Procalcitonin.

Our analysis of peripheral blood cell counts in COVID-19 patients reveals that the severe group shows significantly elevated white blood cell (WBC) and neutrophil counts compared to the mild group. In contrast, lymphocyte and monocyte counts are markedly reduced. Despite these differences, both groups maintain WBC and monocyte counts (M) within the normal physiological range (WBC: 3.5-9.5×10^9^/L, M: 0.1-0.6×10^9^/L). However, in the severe group, the neutrophil count (N) exceeds the upper limit of the normal range, while the lymphocyte count (L) falls below the lower limit of the reference range (N: 1.8-6.3×10^9^/L, L: 1.1-3.2×10^9^/L), as shown in **Figure 3A**. In COVID-19 patients without co-infections, neutrophil and monocyte counts remain within the normal reference range, whereas lymphocyte counts fall below the lower limit. Notably, lymphocyte counts in severe patients are significantly lower than those in mild patients (**Figure 3B**). These findings suggest that SARS-CoV-2 infection may suppress the host immune system’s antiviral response by reducing lymphocyte populations. Among co-infected patients, neutrophil counts are significantly higher than those in non-co-infected patients, while lymphocyte counts show a marked decline. Monocyte counts in co-infected patients exhibit an increasing trend, although this difference is not statistically significant (**Figure 3C**). Additionally, in both mild and severe patients, the co-infected group demonstrates significantly elevated neutrophil counts compared to the non-co-infected group. However, no significant differences in lymphocyte counts are observed between these groups, as shown in **Figure 3D**. These results indicate that co-infections substantially affect neutrophil counts in COVID-19 patients, whereas their impact on lymphocyte counts appears to be comparatively minimal.

Nearly all COVID-19 patients exhibit elevated inflammatory markers, including IL-6, hs-CRP, SAA, and PCT, exceeding normal reference ranges (IL-6: 0-7 pg/mL, hs-CRP: 0-3 mg/L, SAA: 0-6.4 mg/L, PCT: 0-0.046 ng/mL). Serum levels of these markers were significantly higher in the severe group compared to the mild group (**Figure 3E**). Among patients without co-infections, inflammatory marker levels in the severe group were markedly elevated compared to the mild group (**Figure 3F**). Patients with co-infections showed significantly higher levels of inflammatory markers than those non-co-infections (**Figure 3G**). In mild cases, inflammatory marker levels in the co-infected group were moderately elevated compared to the non-co-infected group. In severe cases, the co-infected group exhibited significantly higher levels of inflammatory markers than the non-co-infected group (**Figure 3H**). These findings suggest that COVID-19 infection leads to elevated inflammatory markers, and co-infections further amplify these inflammatory responses, exacerbating disease progression.

### 3.3 Levels of coagulation biomarkers in patients with COVID-19

**Figure 4:**
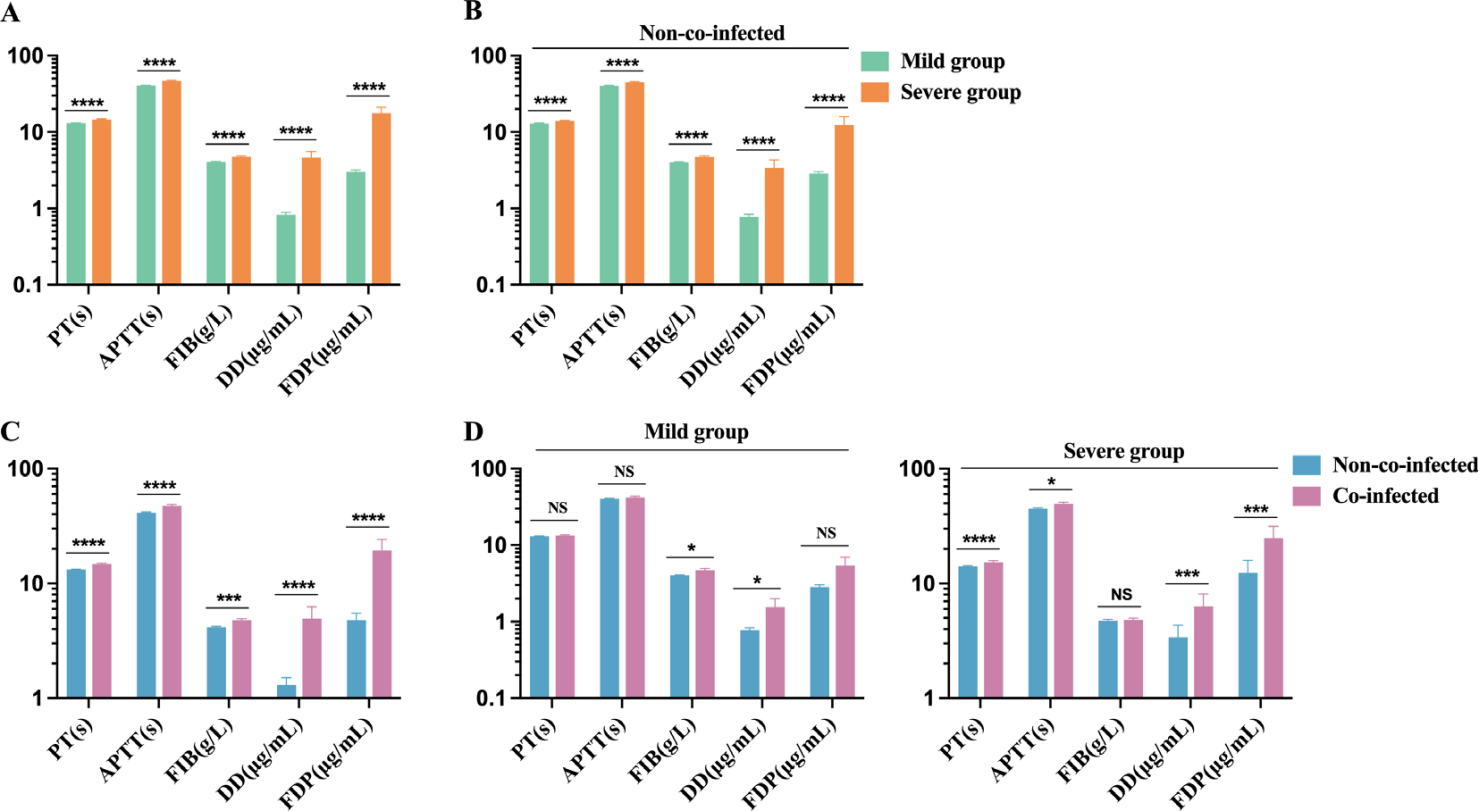
Comparison of coagulation function biomarker levels in COVID-19 patients. **(A)** Severe group (n=173) VS. Mild group (n=431); **(B)** Severe group (n=100) VS. Mild group (n=402) in non-co-infected patients; **(C)** Co-infected group (n=102) VS. Non-co-infected group (n=502); **(D)** Co-infected group VS. Non-co-infected group in the mild (co-infected: n=29, non-co-infected: n=402) and severe groups (co-infected: n=73, non-co-infected=100), respectively. n: Total number of patients with available data. All data presented as the mean ± SEM. Differences were tested using unpaired t test. P<0.05 was considered significant. *P<0.05, **P<0.01, ***P<0.001, ****P<0.0001; NS, not significant. PT, prothrombin time; APTT, activated prothrombin time; FIB, Fibrinogen; DD, D-Dimer; FDP, Fibrinogen degradation products.

The biomarkers for coagulation function in the severe group include PT, APTT, FIB, D-Dimer, and FDP, all of which exceed the upper limit of the normal reference range (PT: 11-14 s, APTT: 28-43.5 s, FIB: 2-4 g/L, D-Dimer: 0-0.5 μg/mL, and FDP: 0-5 μg/mL). These markers are significantly elevated compared to those in the mild group, as shown in **Figure 4A**. Among non-co-infected COVID-19 patients, coagulation function markers in the severe group were notably higher than those in the mild group, where certain markers also showed mild increases (**Figure 4B**). Furthermore, coagulation function markers in the co-infected group were substantially higher than in the non-co-infected group, as demonstrated in **Figure 4C**. In both mild and severe cases, the co-infected group exhibited varying degrees of elevation in coagulation function markers compared to the non-co-infected group (**Figure 4D**). These findings suggest that COVID-19 infection is associated with significant increases in coagulation function markers, and that co-infections further amplify this elevation, contributing to the development of coagulation dysfunction.

### 3.4 Biomarkers of myocardial injury in patients with COVID-19

**Figure 5:**
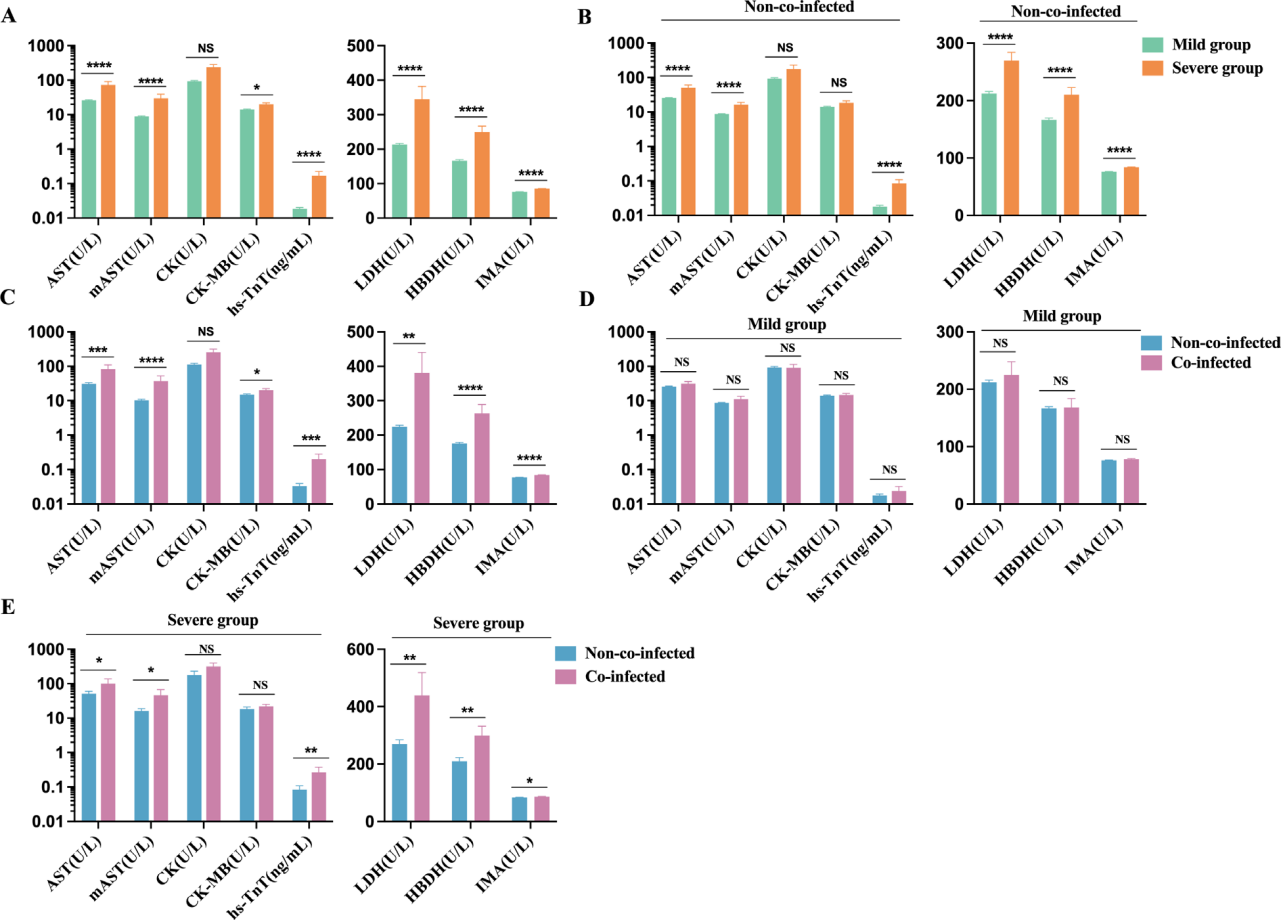
Comparative analysis of myocardial injury biomarkers in COVID-19 patients. **(A)** Severe group (n=115 for hs-TnT, n=132 for AST, m-AST, CK, CK-MB, LDH, HBDH, and IMA) VS. Mild group (n=217 for hs-TnT, n=291 for AST, m-AST, CK, CK-MB, LDH, HBDH, and IMA); **(B)** Severe group (n=60 for hs-TnT, n=73 for AST, m-AST, CK, CK-MB, LDH, HBDH, and IMA) VS. Mild group (n=197 for hs-TnT, n=269 for AST, m-AST, CK, CK-MB, LDH, HBDH, and IMA) in non-co-infected patients; **(C)** Co-infected group (n=75 for hs-TnT, n=81 for AST, m-AST, CK, CK-MB, LDH, HBDH, and IMA) VS. Non-co-infected group (n=257 for hs-TnT, n=342 for AST, m-AST, CK, CK-MB, LDH, HBDH, and IMA); **(D)** Co-infected group VS. Non-co-infected group in the mild (co-infected: n=20 for hs-TnT, n=22 for AST, m-AST, CK, CK-MB, LDH, HBDH, and IMA, non-co-infected: n=197 for hs-TnT, n=269 for AST, m-AST, CK, CK-MB, LDH, HBDH, and IMA) and **(E)** the severe groups (co-infected: n=55 for hs-TnT, n=59 for AST, m-AST, CK, CK-MB, LDH, HBDH, and IMA, non-co-infected: n=60 for hs-TnT, n=73 for AST, m-AST, CK, CK-MB, LDH, HBDH, and IMA), respectively. n: Total number of patients with available data. All data presented as the mean ± SEM. Differences were tested using unpaired t test. P<0.05 was considered significant. *P<0.05, **P<0.01, ***P<0.001, ****P<0.0001; NS, not significant. hs-TnT, Hypersensitive cardiac troponin; AST, aspartate aminotransferase; m-AST, mitochondrialaspartate aminotransferase; CK, creatine kinase; CK-MB, Creatine kinase isoenzyme MB; LDH, lactic dehydrogenase; HBDH, α Hydroxybutyrate dehydrogenase; IMA, Ischemic modified albumin.

We observed that, compared to the mild group, the severe group had significantly elevated levels of AST, m-AST, hs-TnT, LDH, HBDH, and IMA, all exceeding the upper limits of the reference range (AST: 15-40 U/L; m-AST: 0-15 U/L; hs-TnT: 0-0.014 ng/mL; LDH: 120-250 U/L; HBDH: 72-182 U/L; IMA: 0-85 U/mL). CK and CK-MB levels showed a trend toward the upper limit of the reference range (CK: 50-310 U/L; CK-MB: 0-25 U/L), as depicted in **Figure 5A**. In non-co-infected COVID-19 patients, the severe group exhibited significantly higher levels of AST, m-AST, hs-TnT, LDH, HBDH, and IMA compared to the mild group. CK and CK-MB levels in the severe group showed an upward trend, but the increase was not statistically significant. In the mild group, hs-TnT levels exceeded the upper reference limit (**Figure 5B**), suggesting that COVID-19 infection can induce myocardial injury, thereby exacerbating disease progression. Furthermore, in the co-infected group, levels of AST, m-AST, hs-TnT, CK-MB, LDH, HBDH, and IMA were significantly higher than those in the non-co-infected group, while CK levels showed an upward trend (**Figure 5C**). Among mild patients, no significant difference in cardiac injury biomarkers was observed between co-infected and non-co-infected groups, although a tendency toward elevation was noted in the co-infected group (**Figure 5D**). In severe patients, the co-infected group had markedly higher levels of cardiac injury biomarkers compared to the non-co-infected group (**Figure 5E**). These findings suggest that co-infections in COVID-19 patients may heighten the risk of myocardial injury, particularly in those with severe disease manifestations.

### 3.5 Anemia and impaired protein synthesis in COVID-19 patients

**Figure 6:**
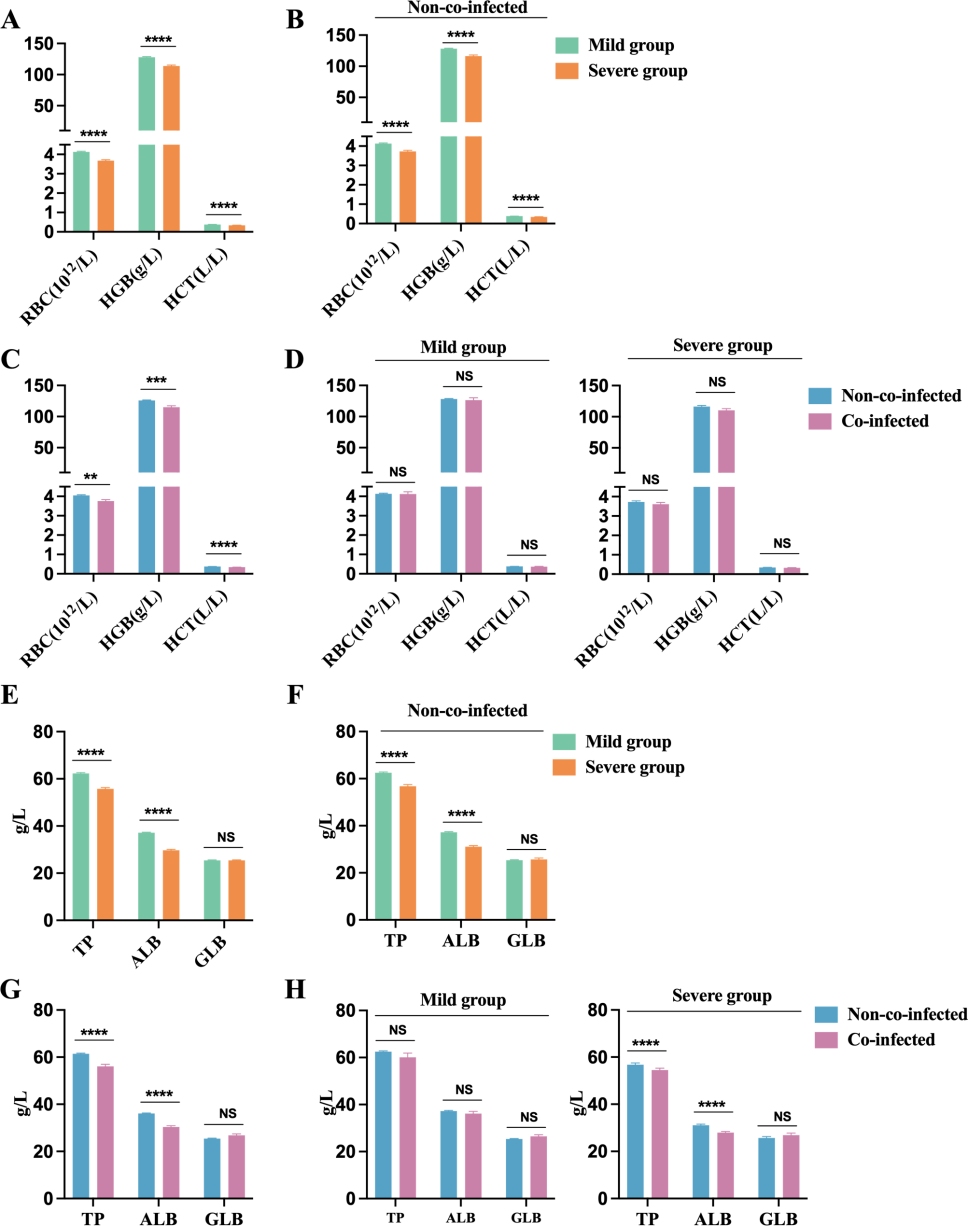
Anemia and impaired protein synthesis in COVID-19 patients. Anemia biomarkers: **(A)** Severe group (n=454) VS. Mild group (n=176); **(B)** Severe group (n=454) VS. Mild group (n=176) in non-co-infected patients; **(C)** Co-infected group (n=106) VS. Non-co-infected group (n=524); **(D)** Co-infected group VS. Non-co-infected group in the mild (co-infected: n=31, non-co-infected: n=423) and severe groups (co-infected: n=75, non-co-infected=101), respectively. Protein synthesis biomarkers: **(E)** Severe group (n=167) VS. Mild group (n=435); **(F)** Severe group (n=94) VS. Mild group (n=405) in non-co-infected patients; **(G)** Co-infected group (n=103) VS. Non-co-infected group (n=499); **(H)** Co-infected group VS. Non-co-infected group in the mild (co-infected: n=30, non-co-infected: n=405) and severe groups (co-infected: n=73, non-co-infected=94), respectively. n: Total number of patients with available data. All data presented as the mean ± SEM. Differences were tested using unpaired t test. P<0.05 was considered significant. *P<0.05, **P<0.01, ***P<0.001, ****P<0.0001; NS, not significant. RBC, Red blood cell count; Hb, Hemoglobin; HCT, hematokrit; TP, Total protein; ALB, Albumin; GLB, Globulin.

We noted that the severe group exhibited dramatically lower red blood cell counts (RBC), hemoglobin (HGB), and hematocrit (HCT) compared to the mild group, all falling below the minimum reference range (RBC: 4.3-5.8×10¹²/L, HGB: 130-175 g/L, HCT: 0.40-0.50 L/L), as shown in **Figure 6A**. Among non-co-infected COVID-19 patients, RBC, HGB, and HCT levels were all below the minimum reference range, with significantly lower levels observed in the severe group compared to the mild group (**Figure 6B**). This suggests that COVID-19 infection may contribute to the development of anemia in these patients. Patients with co-infections exhibited significantly lower RBC, HGB, and HCT values compared to non-co-infected patients, as shown in **Figure 6C**. In both mild and severe cases, no significant differences in red blood cell parameters were observed between the co-infected and non-co-infected groups. However, both groups showed values that remained below the lower limit of the reference range (**Figure 6D**).

In COVID-19 patients, TP and ALB levels drop below the lower limit of the reference range (TP: 65-85 g/L, ALB: 40-55 g/L), while globulin (GLB) levels remain within the reference range (20-40 g/L). The reduction in total protein primarily results from decreased albumin levels. Patients in the severe group exhibit significantly lower TP and ALB levels compared to those in the mild group, as shown in **Figure 6E**. Among non-co-infected COVID-19 patients, the severe group demonstrated significantly lower TP and ALB levels than the mild group, as illustrated in **Figure 6F**. Additionally, patients with co-infections exhibited significantly lower TP and ALB levels compared to those without co-infections, as shown in **Figure 6G**. In severe cases, TP and ALB concentrations were notably lower in patients with co-infections than in those non-co-infections, as illustrated in **Figure 6H**. These findings suggest that co-infections in COVID-19 patients may exacerbate reductions in host protein levels, contributing to malnutrition and potentially anemia. It is crucial for healthcare providers to ensure adequate nutritional support for critically ill patients during their treatment.

### 3.6 Correlation among inflammatory factors, coagulation function, and myocardial injury biomarkers in COVID-19 patients

**Figure 7:**
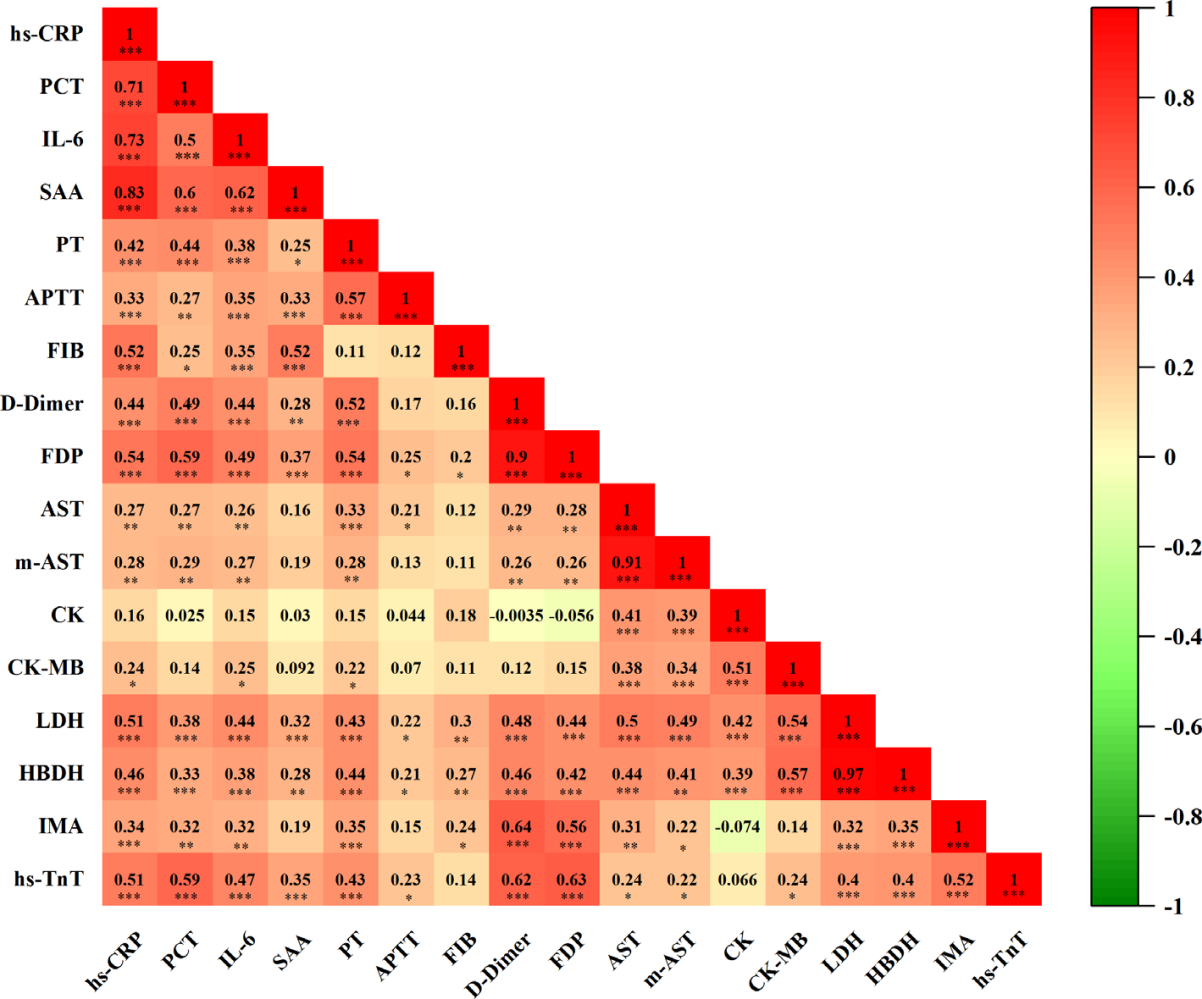
Correlation analysis of inflammatory factors, coagulation function, and biomarkers of myocardial injury(n=101). Correlation matrix results of Spearman correlations, visualized by correlation coefficients (r) as a heat map alongside with P value. In the heat map, the color blocks are determined by correlation values. Red indicates positive correlation, while green signifies negative correlation. The intensity of the color correlates directly with the strength of the correlation. n: Total number of patients with available data. P<0.05 was considered significant. *P<0.05, **P<0.01, ***P<0.001.

As shown in **Figure 7**, we conducted a correlation analysis to investigate the interconnection between inflammatory response, coagulopathy, and myocardial damage in COVID-19 patients. The results revealed that inflammatory factors were positively correlated with coagulation function markers. Moreover, inflammatory factors were positively associated with cardiac injury biomarkers, and coagulation function markers also showed a positive correlation with cardiac injury biomarkers. In conclusion, cardiovascular biomarkers in COVID-19 patients are strongly correlated with inflammatory factors and coagulation function markers, suggesting potential cardiac involvement driven by inflammation and coagulopathy in these patients.

## 4 Discussion

The results of this study indicate a higher prevalence of severe COVID-19 disease among males compared to females, with older age groups being predominantly affected, consistent with prior research. Severe cases among older males may result from diminished immune responses and a higher prevalence of underlying conditions, which increase susceptibility to SARS-CoV-2 infection (16). Research shows that 94.2% of severe COVID-19 patients were simultaneously infected with multiple microorganisms, including viruses, bacteria, and fungi (17). Our findings further suggest that severe COVID-19 patients experience prolonged recovery times and are more prone to co-infections, with antibiotic-resistant cases exhibiting a notable incidence of mixed infections. In contrast, co-infections among mild patients were relatively uncommon, underscoring the need for targeted care in severe cases where co-infections may exacerbate disease severity.

SARS-CoV-2 infection can rapidly activate both non-specific and specific immune responses. Neutrophils play a key role in the non-specific inflammatory response by releasing inflammatory mediators, while lymphocytes are crucial for the specific immune response (18). When the specific immune response fails to effectively eliminate the virus, the non-specific inflammatory response may intensify, resulting in tissue damage, hypoxia, and necrosis. This unchecked response can lead to a cytokine storm, causing acute lung injury and respiratory distress syndrome (19, 20). Imbalanced immune responses and weakened adaptive immunity are major contributors to severe inflammatory reactions in COVID-19. Peripheral blood leukocyte counts, neutrophil counts, and lymphocyte counts serve as indicators of inflammatory and immune status and may help predict disease severity in COVID-19 patients (21). We observed a significant reduction in lymphocyte counts among patients with COVID-19, along with an increase in neutrophil counts in individuals presenting with co-infections. Both neutrophilia and lymphopenia are closely linked to a heightened inflammatory state and cytokine storm, integral components of the pathogenic mechanisms of COVID-19 (22, 23).

COVID-19 progression is often mild in its initial stages but can progress to more severe stages over time. SARS-CoV-2 infection rapidly activates T cells, leading to the release of pro-inflammatory cytokines such as granulocyte-macrophage colony-stimulating factor (GM-CSF) and IL-6. GM-CSF, in turn, activates inflammatory granulocytes and macrophages, initiating an inflammatory cascade that contributes to severe inflammation and immune-mediated damage in the lungs and other organs (24–26). Elevated levels of inflammatory markers such as IL-6, CRP, and serum amyloid A (SAA) have been observed in most patients who succumbed to the disease, underscoring the central role of the inflammatory response (22). One study reported that 52% of COVID-19 patients exhibited elevated IL-6 levels, while 86% showed increased CRP, reflecting a significant inflammatory state (24). IL-6 is a strongly pro-inflammatory cytokine, a major trigger of cytokine storms, and a critical component of the acute inflammatory cascade (26). SAA can promote inflammatory responses directly or indirectly through various pathways, such as activating inflammatory cells and inducing the release of inflammatory factors by interacting with HDL to promote chemotaxis (27). Procalcitonin (PCT) serves as an important marker of systemic inflammatory response, with levels rapidly rising in response to inflammatory stimuli, particularly in bacterial infections or sepsis (28). Several studies have demonstrated a positive correlation between elevated PCT levels and COVID-19 severity (13, 25). Our analysis revealed significantly higher serum levels of IL-6, CRP, SAA, and PCT in severe COVID-19 patients compared to mild cases, indicating a strong association with disease severity. Moreover, COVID-19 patients with co-infections exhibited even higher levels of IL-6, CRP, and PCT, suggesting that bacterial co-infections may amplify the inflammatory response and worsen patient outcomes.

The role of IL-6 in the pathophysiology of anemia in chronic diseases is significant (29). IL-6 suppresses the erythropoietin response and erythrocyte survival, while also inhibiting erythropoiesis and hemoglobin synthesis in the bone marrow. Our findings revealed that erythrocyte counts, hemoglobin levels, and hematocrit levels were significantly lower in the severe group than those in the mild group, all falling below the lower limit of the reference range. This suggests a predisposition to anemia in severe COVID-19 patients, likely due to inflammatory anemia caused by heightened inflammation. Additionally, these parameters were lower in co-infected patients than in non-co-infected individuals. We also observed that patients with severe infections experience impaired protein synthesis and are prone to hypoalbuminemia. This condition occurs more frequently in the later stages of infection and is linked to decreased synthesis and increased catabolism of serum proteins, alongside heightened capillary permeability induced by the inflammatory response to COVID-19 (30). The rapid decline in serum albumin levels during the acute phase of infection reflects the severity of the systemic inflammatory response and serves as a widely used indicator of poor prognosis in critical care settings (31).

COVID-19 has a complex interplay with the body’s coagulation system, where inflammation activates coagulation, and coagulation, in turn, amplifies inflammation. Elevated levels of IL-6 and IL-1 have been shown to increase coagulation markers such as D-Dimer, creating a feedback loop between inflammation and coagulation (32, 33). Approximately 20% of COVID-19 patients exhibit coagulation abnormalities, particularly in severe cases. In these patients, hypoxia and tissue damage activate coagulation pathways, leading to microthrombi formation and prolonged prothrombin time (PT) (34). This disruption is further evidenced by elevated fibrinogen (FIB) levels, which serve as both a coagulation factor and an inflammation biomarker (35, 36). In severe cases, significantly increased fibrin degradation products indicate hyperfibrinolysis and impaired coagulation function (37). Consistent with prior studies, our findings confirm that critically ill COVID-19 patients experience substantial coagulation dysfunction, which is strongly associated with poor clinical outcomes.

The cytokine storm induced by SARS-CoV-2 causes vascular endothelial damage, hypercoagulability, myocardial inflammation, and subsequent myocardial injury (38). Myocardial injury is a significant risk factor for in-hospital mortality among critically ill COVID-19 patients. Severe SARS-CoV-2 infection increases the risk of acute coronary syndromes, which often lead to myocardial injury (39), a major contributor to disseminated intravascular coagulation (DIC). Tang et al. (40) demonstrated that abnormal coagulation parameters, particularly elevated plasma D-Dimer and fibrin degradation products (FDP), are prevalent among COVID-19 patients who succumbed to the disease. When DIC occurs, vascular endothelial damage and the release of inflammatory factors, such as IL-6 and TNF-α, stimulate the release of large quantities of tissue factors from the vascular endothelium. This cascade creates a hypercoagulable state, leading to extensive microthrombi formation in the cardiac microcirculation and subsequent myocardial injury. Clinical data from COVID-19 patients revealed elevated levels of lactate dehydrogenase (LDH) and creatine kinase-MB (CK-MB), with troponin levels also elevated in some severe cases (41). These elevated cardiac injury markers were associated with myocardial injury, disease severity, and poor prognosis (42). In this study, we found that in the mild group of non-co-infected COVID-19 patients, hs-TnT levels exceeded the upper limit of the reference range. Biomarkers of myocardial injury were significantly elevated in the co-infected group compared to the non-co-infected group, particularly in severely ill patients. Cardiac troponin, a sensitive and specific indicator of myocardial injury, serves as a reliable measure of cardiovascular risk. Elevated hs-TnT levels at the time of consultation in SARS-CoV-2-infected patients indicate that the virus can impair myocardial function. The markedly high hs-TnT levels observed in severe cases suggest that myocardial injury is a key risk factor for COVID-19 exacerbation. Our correlation analysis showed that hs-TnT levels in COVID-19 patients were significantly and positively correlated with inflammatory factors and coagulation markers. This finding suggests that thrombotic inflammation is a major contributor to SARS-CoV-2-mediated myocardial injury. When hs-TnT levels are abnormally elevated, prompt intervention is necessary, and vigilance is required to mitigate the risk of thrombotic inflammation.

There are several limitations to our study. First, this was a single-center observational cohort study with a limited number of cases and small sample sizes for some parameters, which restricts the generalizability of the findings and is insufficient to draw fully reliable conclusions. Further cohort studies are needed for validation. Second, not all participants had complete data for markers related to inflammation, coagulation, and cardiac injury throughout their hospitalization. Incomplete data collection across multiple time points may have introduced confounding effects in analyzing the relationship between cardiac injury markers and those related to inflammation and coagulation in COVID-19 patients. Third, as this was a retrospective analytical study, data collection posed challenges and might not have accounted for all potential confounding factors, which could introduce bias despite rigorous data collection and analysis. Fourth, while this study excluded patients admitted primarily for heart disease, it did not exclude those with a history of heart disease. Considering that COVID-19 infection can induce abnormalities in coagulation function and cardiac injury biomarkers, the inclusion of patients with a history of cardiac conditions might have influenced the results. Finally, since all participants in this study were hospitalized patients, the findings may not be fully applicable to all individuals infected with SARS-CoV-2, such as outpatients or those with mild symptoms in the general population.

In summary, with the gradual understanding of COVID-19, it is now recognized not only as interstitial pneumonia but also as a vascular disease. Co-infections in COVID-19 patients can trigger severe inflammatory responses. This excessive inflammation may lead to coagulation disorders and myocardial injury, all of which are key contributors to disease progression and deterioration. Therefore, implementing infection prevention measures to minimize the spread of co-infections among hospitalized COVID-19 patients is essential.

## Data Availability

The original contributions presented in the study are included in the article/supplementary material. Further inquiries can be directed to the corresponding author.
